# Developmental patterns of extracellular matrix molecules in the embryonic and postnatal mouse hindbrain

**DOI:** 10.3389/fnana.2024.1369103

**Published:** 2024-03-01

**Authors:** Ildikó Wéber, Adél Dakos, Zoltán Mészár, Clara Matesz, András Birinyi

**Affiliations:** ^1^Laboratory of Brainstem Neuronal Networks and Neuronal Regeneration, Department of Anatomy, Histology, and Embryology, Faculty of Medicine, University of Debrecen, Debrecen, Hungary; ^2^Department of Pediatric and Preventive Dentistry, Faculty of Dentistry, University of Debrecen, Debrecen, Hungary; ^3^Division of Oral Anatomy, Faculty of Dentistry, University of Debrecen, Debrecen, Hungary

**Keywords:** hyaluronan, proteoglycan, tenascin, link protein, quantitative analysis

## Abstract

Normal brain development requires continuous communication between developing neurons and their environment filled by a complex network referred to as extracellular matrix (ECM). The ECM is divided into distinct families of molecules including hyaluronic acid, proteoglycans, glycoproteins such as tenascins, and link proteins. In this study, we characterize the temporal and spatial distribution of the extracellular matrix molecules in the embryonic and postnatal mouse hindbrain by using antibodies and lectin histochemistry. In the embryo, hyaluronan and neurocan were found in high amounts until the time of birth whereas versican and tenascin-R were detected in lower intensities during the whole embryonic period. After birth, both hyaluronic acid and neurocan still produced intense staining in almost all areas of the hindbrain, while tenascin-R labeling showed a continuous increase during postnatal development. The reaction with WFA and aggrecan was revealed first 4th postnatal day (P4) with low staining intensities, while HAPLN was detected two weeks after birth (P14). The perineuronal net appeared first around the facial and vestibular neurons at P4 with hyaluronic acid cytochemistry. One week after birth aggrecan, neurocan, tenascin-R, and WFA were also accumulated around the neurons located in several hindbrain nuclei, but HAPLN1 was detected on the second postnatal week. Our results provide further evidence that many extracellular macromolecules that will be incorporated into the perineuronal net are already expressed at embryonic and early postnatal stages of development to control differentiation, migration, and synaptogenesis of neurons. In late postnatal period, the experience-driven neuronal activity induces formation of perineuronal net to stabilize synaptic connections.

## 1 Introduction

The extracellular matrix (ECM) fills the spaces among neurons and glial cells and plays a crucial role in normal brain development (Cragg, 1979; Nicholson and Syková, 1998). Traditionally viewed as a scaffolding structure, the ECM has been found to have multiple functions in the central nervous system (CNS). It acts as a physical barrier and limits ion and water diffusion and cell movements. Additionally, it binds growth factors and concentrates them on receptors of developing neurons and glial cells. The ECM molecules also activate intracellular signaling pathways and interact with cell surface receptors and soluble factors. Mutations in ECM-related genes and knockout experiments in animals have highlighted the ECM’s importance in brain development (Maeda, 2015). The ECM in the CNS consists of different families of molecules, including glycosaminoglycans (GAGs) like hyaluronic acid, proteoglycans, and glycoproteins such as tenascins and link proteins.

*Hyaluronic acid* also called *hyaluronan* (HA) is a major component of the brain’s extracellular matrix. It is synthesized in astrocytes and neurons and secreted into the extracellular space where it forms a scaffold for binding proteoglycans through hyaluronic acid and proteoglycan link proteins (Itano et al., 1999; Itano and Klimata, 2002; Lau et al., 2013). Besides its physical properties, HA interacts with cell surface receptors where it is involved in the regulation various cellular functions (Nagy et al., 1995; Casini et al., 2010; Dzwonek and Wilczynski 2015). The distribution and concentration of hyaluronan change during brain development and it is involved in movements of neural crest cell, or proliferation, differentiation, migration of neural progenitor cells (Kazanis and ffrench-Constant, 2011; Preston and Sherman, 2011; Casini et al., 2012; Su et al., 2017). Perturbations in the concentration of extracellular HA can change the physical characteristics of the ECM and affect the morphological and physiological properties of the developing neocortex. Increasing the stiffness of the ECM leads to movements of cells towards this area (Lange and Fabry, 2013; Kim et al., 2018). Loosening the ECM facilitates the migration of developing neurons and creates a permissive environment for the formation of early cortical folds in the developing human neocortex (Long et al., 2018). Alterations in hyaluronan levels can impact neuronal network excitability (Wilson et al., 2020) which may lead to neurodevelopmental disorders (Gao and Penzes, 2015; Balashova et al., 2019; Mukhina, 2019). The HA was also proven to regulate the formation and function of excitatory synapses in developing neuronal networks (Wilson et al., 2020).

*Proteoglycans,* composed of glycosaminoglycan chains linked to a core protein, are the major components of the brain ECM (Kjellén and Lindahl, 1991). In the brain, chondroitin sulfate proteoglycans (CSPGs) are abundant, with lecticans being the most prevalent. CSPGs are transiently expressed in high levels in certain areas of the developing brain where they play important roles in almost all steps of neuronal development. They control the proliferation and differentiation of neuronal stem cells (Margolis and Margolis 1989; Kabos et al., 2004; Götz and Huttner, 2005; Mencio et al., 2021), as elimination of CSPGs at early stages of development resulted in decreased proliferation of neuronal progenitors and increased number of astrocytes in the mouse neocortex (Sirko et al., 2007; Tham et al., 2010). They also can act as barriers or attractants for migrating cells, depending on the context (Perris et al., 1991; Faissner et al., 1994; Landolt et al., 1995; Clement et al., 1999), as well as regulating the axon outgrowth (Bicknese et al., 1994; Friedlander et al., 1994; Pierres-Neto et al., 1998; Mencio et al., 2021). The CPGS are responsible for laminar organization in the cortex, and they are also involved in neuronal aggregation (Schwartz and Domowicz, 2004) by interacting with morphogenic molecules such as semaphorins (Zhu et al., 1999; Zimmer et al., 2010) or activating pleiotropin—PTPϛ signaling pathway (Maeda and Noda, 1998). These data suggest that proteoglycan molecules cannot be regarded as simple supportive or inhibitory factors during different stages of embryonic development, but their effects are highly dependent on their spatial and temporal expression, specific sulfation motifs as well as their interaction with cell surface receptors and different growth factors.

*Tenascins*, characterized by specific common molecular structures, are expressed in developing neural tissues (Faissner, 1997) and have diverse functions in neurogenesis and neuronal process formation (Bartsch et al., 1992; Riou et al., 1992). Tenascin-C exhibits site-restricted distribution in regions that are active in early embryonic neurogenesis (Bartsch et al., 1992; Riou et al., 1992). The expression of tenascin-R becomes high during the postnatal period when it regulates oligodendrocyte differentiation (Pesheva et al., 1997; Pesheva and Probmeister, 2000), axon fasciculation and myelination (Bartsch et al., 1993; Xiao et al., 1996), and neurogenesis in the postnatal and adult brain (Shaghatelyan et al., 2004; Xu et al., 2014). In later stages of development tenascin-R is mainly associated with myelinated axons at the nodes of Ranvier and also plays a pivotal role in the formation of the perineuronal net (PNN) around the somata, proximal dendrites, and axon initial segments of a certain population of interneurons and motoneurons in the central nervous system (Morawski et al., 2014).

During embryonic development and the postnatal period, the majority of the extracellular matrix molecules remain dissolved in the extracellular space as diffused or interstitial ECM. After birth, however, a small fraction of ECM molecules form specialized structures around neuronal cell bodies, proximal dendrites and axon initial segments as perineuronal net or enwraps synapses as a perisynaptic matrix as well as the node of Ranvier as a perinodal matrix (Deepa et al., 2006; Brückner et al., 2008; Fawcett et al., 2019). PNNs primarily consist of hyaluronan, lecticans, HAPLN link proteins, and tenascin-R (Giamanco et al., 2010; Kwock et al., 2010; Morawski et al., 2014) that interact with each other (Brückner et al., 2000; Bekku et al., 2012; Wang and Fawcett, 2012; Bosiacki et al., 2019). The organization of extracellular molecules into PNNs requires appropriate stimuli and occurs mostly during the critical periods when the experiences from the environment deeply affect synapse refinements (Dityatev et al., 2007; Mcrea et al., 2007; Ye and Miao, 2013). The level of aggrecan was proved to be regulated by sensory experience, therefore it is supposed that activity-dependent production of aggrecan could be the stimulus for the establishment of PNNs (Kind et al., 2013). In aggrecan knockout mice, the ocular dominance plasticity was retained, and the critical period was extended (Rowlands et al., 2018). Chondroitin sulfate proteoglycans could be responsible for both the onset and termination of the critical period, and they also play a pivotal role in the wiring of neuronal circuits (Hensch, 2004; Hou et al., 2017). After its development, the PNNs are involved in the positioning and stabilization of synapses and restrict plasticity in the adult CNS by preventing abnormal synaptic remodeling. As a consequence of these processes, the plasticity of the neural circuits is reduced or lost in many areas of the brain after the critical period and digestion of CSPGs restores neuronal circuits into a juvenile form and induces reactivation of plasticity in different cortical areas (Pizzorusso et al., 2002; Beurdelely et al., 2012; Romberg et al., 2013; Xu et al., 2014).

Through a quantitative analysis, our study investigated the distribution and expression of extracellular matrix molecules in the embryonic and postnatal hindbrain. We found that hyaluronic acid and neurocan were present in high amounts during embryonic development, while versican and tenascin-R were detected in lower intensities. After birth, hyaluronic acid and neurocan continued to exhibit intense staining throughout the hindbrain, while tenascin-R levels increased. We observed the formation of perineuronal nets (PNNs) around neuronal cell bodies, composed mainly of aggrecan and neurocan. HAPLN1 link protein was incorporated into the PNNs during later stages of postnatal development. Considering the limited availability of data on presence and organization of the ECM in the developing brainstem (Heyman et al., 1995; Bekku et al., 2012), our data should also be indispensable to understanding the possible role of ECM molecules in those neuronal circuits of the brainstem which are already established and functioning during embryonic life (e.g., the vestibulo-ocular and vestibulospinal connections).

## 2 Materials and methods

### 2.1 Animals and tissue processing

The experiments were performed on mouse embryos at developing days 13.5, 15.5, and 16.5, (E13.5, E15.5, E16.5), newborns (PO), as well as animals on postnatal days 4, 7, 14 (P4, P7, P14), and adult (4–6 months old) animals (Charles River Laboratory, Strain CD1) according to authorized permission (Registration number: OASZF/822/2011/IT:16.18). After removing from the uterus, the embryos were deeply anesthetized and decapitated. The heads were cut at the midsagittal plane and immersed into Sainte-Marie’s fixative (99% absolute ethanol and 1% glacial acetic acid) for 1 day at 4°C. The postnatal and adult animals were terminally anesthetized with an intraperitoneal injection of 10% urethane (1.3 mL/100 g body weight; Reanal, Budapest, Hungary) then perfused transcardially with physiological saline followed by a Sainte-Marie’s fixative. The brains were removed from the skull and immersed into the same fixative for 1 day at 4°C. After fixation, the specimens were embedded into paraffin and cut sagittally with a microtome at a thickness of 8 μm. The sections were collected on silane-coated slides and left to dry overnight at 37°C. Following deparaffination, the sections were rehydrated, washed in phosphate-buffered saline (PBS), and treated with 3% H_2_O_2_ for 10 min at room temperature.

The study protocol was reviewed and approved by the Animal Care Committee of the University of Debrecen, Hungary according to national laws and EU regulations [European Communities Council Directive of 24 November 1986 (86/609/EEC)] and was properly carried out under the control of the University’s Guidelines for Animal Experimentation (license number: 11/2011/DEMAB).

### 2.2 Histochemistry and immunohistochemistry

Before histochemical and immunohistochemical reactions, all specimens were immersed into 1% bovine serum albumin (BSA) for 30 min for HA and *Wisteria floribunda* agglutinin (WFA) reactions, 1% BSA and 10% normal goat serum for aggrecan, versican, and 3% normal rabbit serum (NRS) for neurocan, tenascin-R, and HAPLN1 reactions.

Hyaluronic acid was detected by applying biotinylated hyaluronan binding protein (bHABP, kindly provided by R. Tammi and M. Tammi, Department of Anatomy, University of Kuopio, Finland, Table 1). The proteoglycan molecules were revealed by using biotinylated *Wisteria floribunda* agglutinin (bWFA, Sigma-Aldrich, Table 1). At the beginning of histochemical procedure, the sections were incubated in a solution of bHABP (1:50) or bWFA (1:500) and dissolved in PBS containing 1% BSA overnight at 4°C. Then the sections were treated with ExtrAvidin Peroxidase Complex (Sigma-Aldrich, St. Louis, Missouri, United States) and reacted with 3,3-diaminobenzidine-tetrahydrochloride (DAB, Sigma-Aldrich) and hydrogen peroxide. After dehydration, the sections were mounted and coverslipped with DPX mounting medium (Sigma-Aldrich, Table 2). In immunohistochemical procedures the following primary antibodies were used (Table 1): rabbit polyclonal anti-aggrecan (1:500), sheep polyclonal anti-neurocan (1:100), goat polyclonal anti-tenascin-R (1:300), rabbit polyclonal anti versican (1:500), and goat polyclonal anti-HAPLN1 (1:300). To increase the exposure of the antigen for aggrecan and versican molecules, the sections were treated with chondroitinase ABC (0.02 U/mL; Sigma-Aldrich) in Tris sodium-acetate buffer, (pH 8) for 1 h at 37°C according to the manufacturer’s instructions. Sections were then treated with primary antibodies dissolved in PBS containing 1% BSA, 3% NGS (for anti-aggrecan and anti-versican), 1%BSA, 3% NRS (for anti-neurocan, anti-tenascin-R, and anti-HAPLN1) overnight at 4°C. The sections were rinsed in PBS, then processed with a solution containing biotinylated goat-anti-rabbit IgG (1:200) for detection of aggrecan and versican, biotinylated rabbit-anti-sheep IgG (1:200) for anti-neurocan, and biotinylated rabbit-anti-goat IgG (1:200) to expose anti-tenascin-R and anti-HAPLN1 (Table 2). After visualizing the immunohistochemical reactions with DAB, the sections were washed in PB, mounted on gelatin-coated slides, and coverslipped.

**Table 1:** Probe, lectin and primary antibodies used for detection of extracellular matrix components.

	Supplier	Species of origin, type	Immunogen	Dilution	Characterization	Controls
Biotinylated hyaluronic acid binding protein (bHABP)	Provided by R. Tammi and M. Tammi, Kuopio, Finland	HA-binding region of aggrecan isolated from bovine articular cartilage.		1:50 (0.2 lg/mL)	By histochemistry (Midura et al., 2003)	HC on rat sternal cartilage
Biotinyilated *Wisteria floribunda* agglutinin (bWFA)	Sigma-Aldrich L1516	Lectin isolated from *Wisteria floribunda*		1:500	By histochemistry (Härtig et al., 1992)	HC on rat sternal cartilage; HC pattern on cerebellum identical to Carulli et al. (2006)
Anti-aggrecan	Merck Millipore AB1031	Rabbit, polyclonal, IgG	GST fusion protein containing amino acids 1,177–1,326 of mouse aggrecan	1:500	By Western blot (Afshari et al., 2010), single band of 60 kDa	WB in our laboratory on rat brain, band of approx. 60 kDa.
Anti-neurocan (mouse/rat)	R&D System AF5800	Sheep, polyclonal IgG	CHO cell derived r.m Neurocan (aa23-637)	1:100	By Western blot (R&D Systems datasheet) at 200 kDa	IHC pattern on rat cerebellum identical to Carulli et al. (2006)
Anti-tenascin R	R&D System, AF 3865	Goat, polyclonal IgG	Mouse myeloma cell line NS0-derived recombinant human tenascin-r isoform1 Glu34-Phe 1,358	1:300	By Western blot in our laboratory, single band of 180kDA.	WB on rat brain and articular cartilage in our laboratory.
Anti-versican (GAG beta domain)	Merck Millipore, AB1033	Rabbit, polyclonal IgG	GST fusion protein containing amino acids 1,360–1,439 of mouse versican	1:500	By Western blot positive control: total 13 days embryo	WB on total 13-day mouse embryo, HC surrounding cartilage.
Anti-hyaluronic acid and proteoglycan link protein1(HAPLN1)	R&D System, AF 2608	Goat, polyclonal IgG	Mouse myeloma cell line NS0-derived recombinant human HAPLN1 Asp16-Asn354	1:300	Used successfully for IHC by Carulli et al. (2006)	IHC pattern on rat cerebellum identical to Carulli et al. (2006)

**Table 2:** Secondary antisera used for detection of extracellular matrix components.

Antisera	Species of origin	Supplier, Cat. no	Dilution
Biotinylated anti-rabbit IgG (aggrecan, versican)	Goat	Vector Laboratories, Burlingame, CA, United States, BA-1000,	1:200
Biotinylated anti-sheep IgG (neurocan)	Rabbit	R&D System Minneapolis, Minnesota, United States, AF5800	1:200
Biotinylated anti-goat IgG (tenascin-R, HAPLN1)	Rabbit	Vector Laboratories, Burlingame, CA, United States, BA-5000	1:200

From each embryonic and postnatal sample, the slides were stained with toluidine blue to identify the location of different nuclei on the sections.

### 2.3 Microscopy and quantitative analysis

Images were captured with the help of an Olympus DP74 camera connected to an Olympus CX31 conventional light microscope. The hindbrains were photographed by using 4X objective with the same setting parameters for all images. The location and the boundaries of different regions and nuclei in the hindbrain were identified in the embryos with the help of “Allen Developing Mouse Brain Atlas”^1^ whereas the “Paxinos and Franklin’s The mouse brain stereotaxic coordinates” was used for the same purpose in postnatal animals (Paxinos and Franklin, 1997).

The staining intensities of different extracellular molecules were measured with the help of ImageJ software.^2^ We performed three different measurements on each slide. First, the intensities were calculated by measuring twenty randomly selected 0.09 μm^2^ areas from those parts of the medulla and pons where the nuclei could not be delineated with the help of the ECM molecules. Then we repeated the same measurements by using areas within different hindbrain nuclei where the extracellular matrix could be easily identified according to its strong cytochemical or immunocytochemical reactions. We collected our data by using three hindbrains from each investigated embryonic and postnatal stage.

To collect the quantitative data for analysis of labeled perineuronal net, the neurons were chosen from six different nuclei including the somatomotor nuclei of the trigeminal and facial nerves, the vestibular nuclear complex, as well as the lateral superior olive and rostral preolivary region. The cells located in these nuclei were strongly labeled with the majority of different ECM molecules at P7, P14, and adult stages. The optical densities of the perineuronal net were measured on photographs captured at a 20X objective lens (NA: 0.5, UPlanFLN, Olympus, Japan). Within each nucleus ten different pixel samples were taken from the PNNs around the neurons and the interstitium among the labeled neurons. The intensity of PNN labeling in the nucleus was calculated as the difference between the average values of these samples. Finally, we measured the background intensities for each ECM molecule and decreased the calculated PNN intensities with this value.

**Figure 1:**
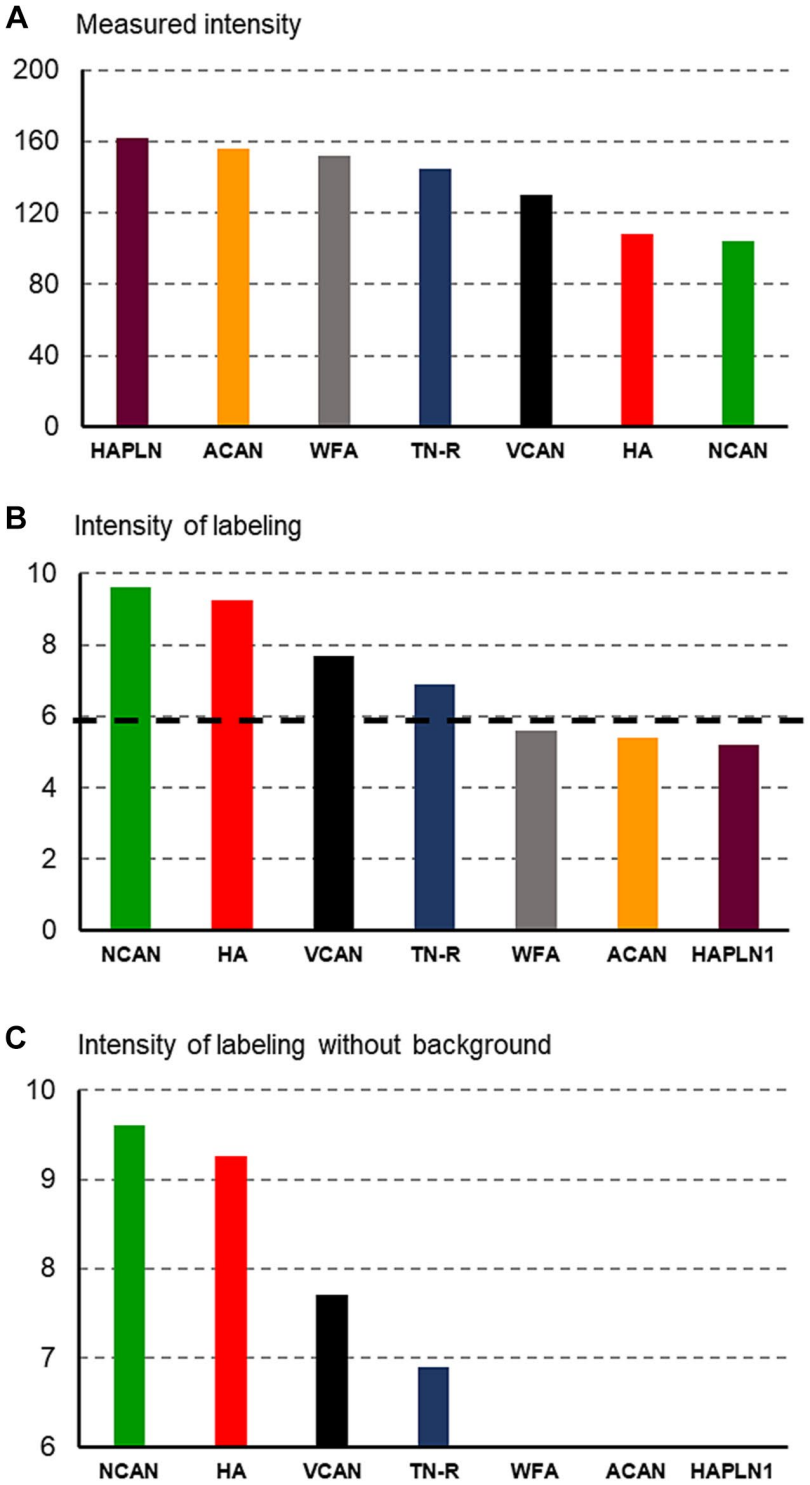
Histograms showing the methods used for quantification of staining intensities of the extracellular molecules. First, the measured intensity values **(A)** were recalculated to be proportional to the concentration of ECM molecules **(B)**, then the background intensity [indicated by a dashed line on **(B)**] was removed from the histograms **(C)**.

**Figure 2:**
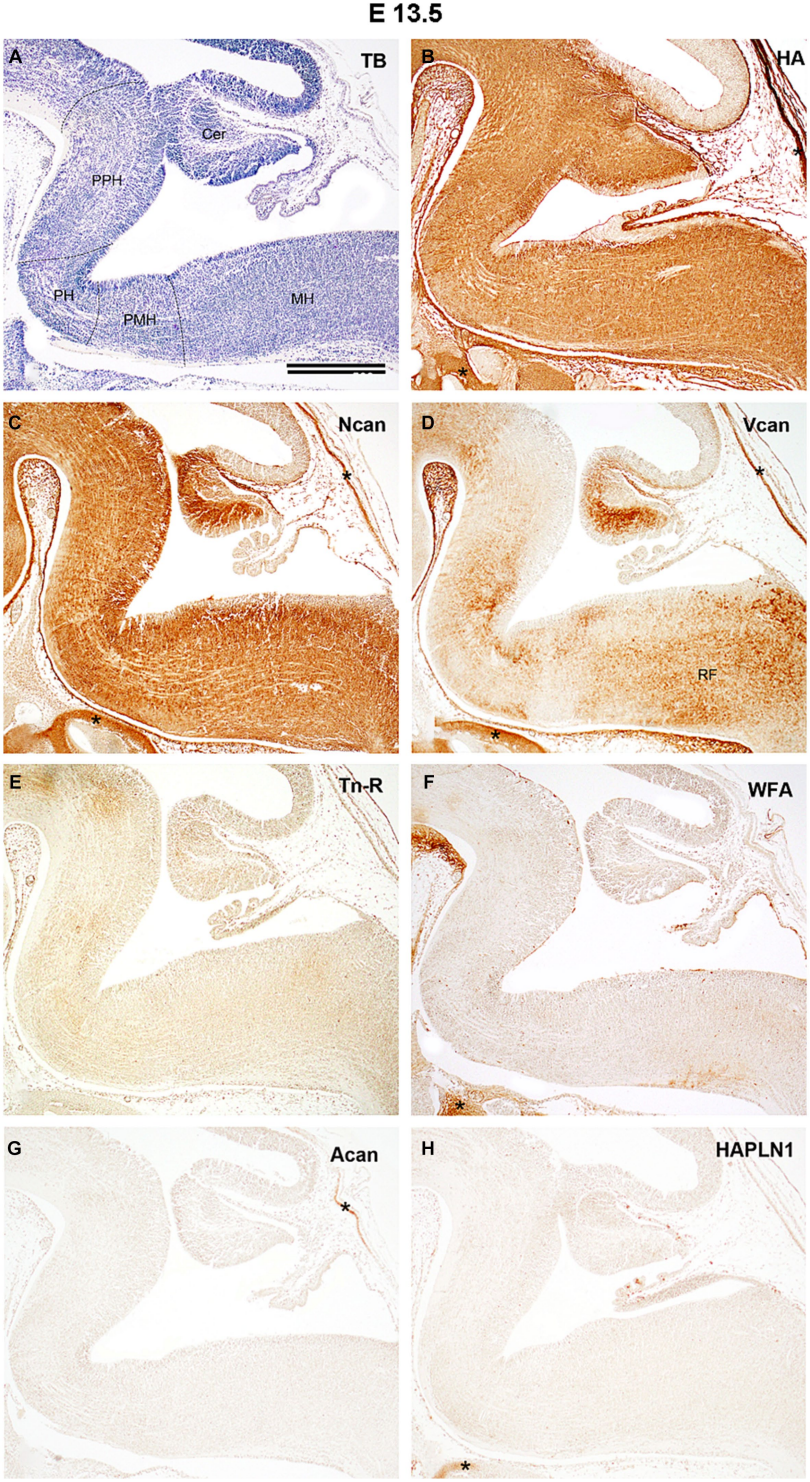
Distribution of extracellular matrix molecules in the hindbrain of developing mouse at embryonic day 13.5. The boundaries between the prepontine (PPH), pontine (PH), pontomedullary (PMH), and medullary (MH) parts of the hindbrain are indicated by dashed lines on the specimen stained with toluidine blue [TB, **(A)**]. Diffuse and evenly distributed interstitial labeling was detected with biotinylated hyaluronic acid binding protein [HA, **(B)**] and antibodies against neurocan [Ncan, **(C)**] whereas versican antibody produces inhomogeneous staining in the hindbrain [Vcan, **(D)**]. No signals were found in slices treated with anti-tenascin-R antibody [Tn-R, **(E)**], biotinylated *Wisteria floribunda* agglutinin [WFA, **(F)**], or with an aggrecan [Acan, **(G)**], and hyaluronic acid and proteoglycan link protein [HAPLN1, **(H)**] antibodies. Asterisks indicate labeled ECM molecules in the cartilage of the skull. Cer, cerebellum; RF, reticular formation. Scale bar 200 μm.

As we revealed the ECM molecules by using DAB histochemistry, the measured intensities were inversely proportional to the concentration of the molecules (Figure 1A). The highest values were taken from those sections where the molecules were not detected (see Figures 1A, 2G,H), whereas the smallest values corresponded to large amounts of substances in the ECM (Figures 1A, 2B,C). To illustrate our data in a way that is widely used in publications, we recalculated our measured values by using the following formula: 1/x × 1,000 where x corresponds to the measured value (Figure 1B). Finally, we removed the background intensities from the figures to emphasize the differences among the intensities of labeling for different ECM molecules (Figure 1C). The background intensity values were calculated by measuring 10 randomly selected areas on those sections where no signals were detected with antibodies against certain ECM molecules (e.g., Figures 2G,H). The statistical analysis was carried out with the IBM SPSS Statistics software (SPSS Inc., Chicago, IL, United States). The mean values of the variables were compared using Mann–Whitney test or Kruskal–Wallis non-parametric analysis of variance.

## 3 Results

The hindbrain or rhombencephalon is the most caudal brain vesicle extending between the cervical and mesencephalic flexures. The hindbrain is divided into a caudal tube-like myelencephalon gives rise to the medulla oblongata and a rostral metencephalon gives origin to the pons and cerebellum. Although as the derivates of the prepontine region the cerebellum could also be regarded as part of the hindbrain, we devoted this article to summarizing our data concerning the distribution of ECM molecules in the embryonic and postnatal medulla and pons. Therefore, in the text we refer to the development of the hindbrain as the developing pons and medulla.

### 3.1 Distribution of ECM molecules in the embryonic hindbrain

By E13.5 the primordium of the cerebellum is located as the upper rhombic lip on the dorsal side of the hindbrain, whereas the ventral hindbrain could be subdivided into prepontine, pontine, pontomedullary, and medullary compartments (Figure 2A). The hyaluronic acid and neurocan molecules appear in high quantity and fill the extracellular space quite homogenously at this age with a notable difference in the ventricular zone of the rhomboid fossa at the pontine flexure since this area was rich in neurocan but poor in HA signals (Figures 2B,C). The third identifiable ECM component, the versican emerges more unevenly in the hindbrain (Figure 2D). The signals produced by other investigated ECM components did not exceed the level of the background intensity (Figures 2E–H). Although large number of nuclei were identified in the hindbrain at this embryonic stage with the help of different methods (e.g., Altman and Bayer, 1980, 1987a,b Allen Developing Mouse Brain Atlas, 2015 Vivian et al., 2017), they could not be revealed by using ECM histology.

**Figure 3:**
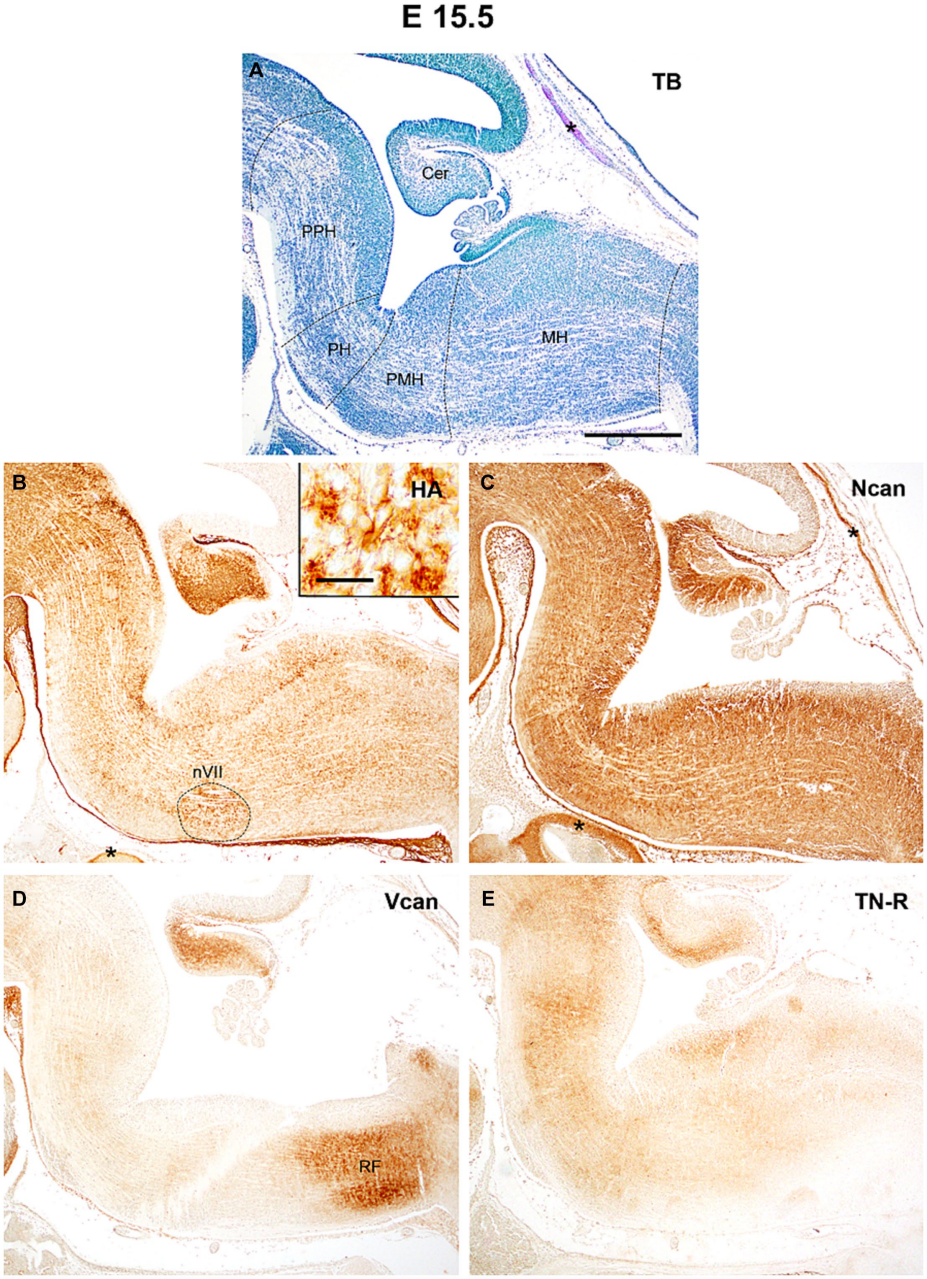
At the age of E15.5 hyaluronic acid (HA) and neurocan (Ncan) immunoreaction shows intense labeling of the neuropil throughout the hindbrain **(B,C)**. Weaker signals were revealed with versican (Vcan) and tenascin-R (TN-R) antibodies **(D,E)**. Insert in **(B)** shows an enlarged area from the facial motor nucleus (nVII). Asterisks indicate metachromasia **(A)** and labeled ECM molecules **(B,C)** in the cartilage of the skull. Cer, cerebellum; PPH, prepontine hindbrain; PH, pontine hindbrain; PMH, pontomedullary hindbrain; MH, medullary hindbrain; RF, reticular formation; TB, toluidine blue. Scale bar low magnification = 200 μm, high magnification = 20 μm.

**Figure 4:**
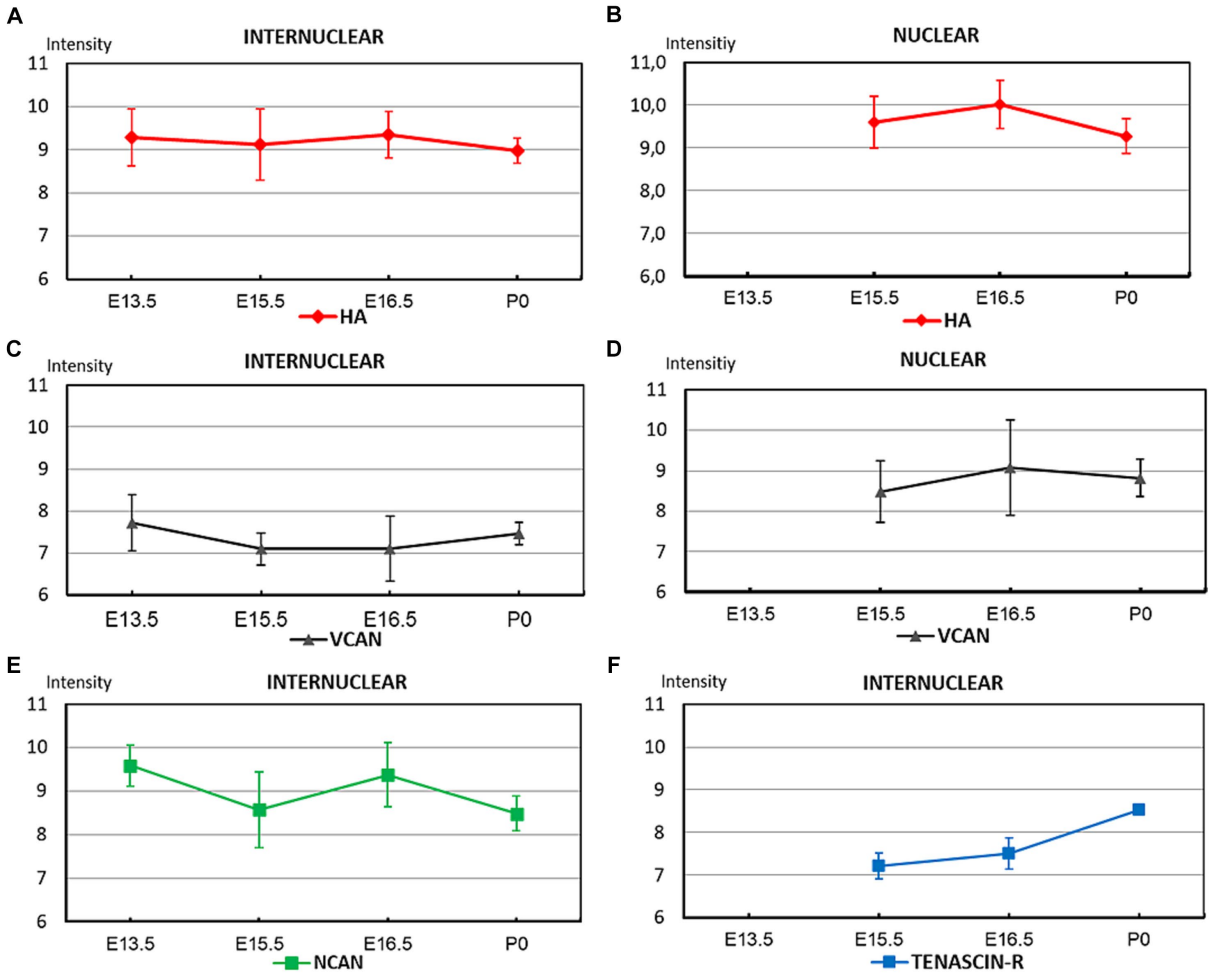
The graphs summarize the optical intensities of ECM molecules in different stages of embryonic development in the internuclear areas **(A,C,E,F)** and in different nuclei **(B,D)** of the hindbrain. The points indicate the average values, the bars correspond to the standard error of the mean.

At E15.5 (Figure 3A) the HA accumulates in the subependymal zone of the prepontine and pontine rhombomeres and circumscribes the facial motor nucleus (Figure 3B). The level versican and neurocan decreased between E13.5 and E15.5 (Figures 3C,D, 4C,E). The versican reaches relatively high level in the reticular formation of the medulla (Figure 3D). The tenascin-R which was not detectable earlier showed homogenous light staining in the interstitium (Figure 3E).

**Figure 5:**
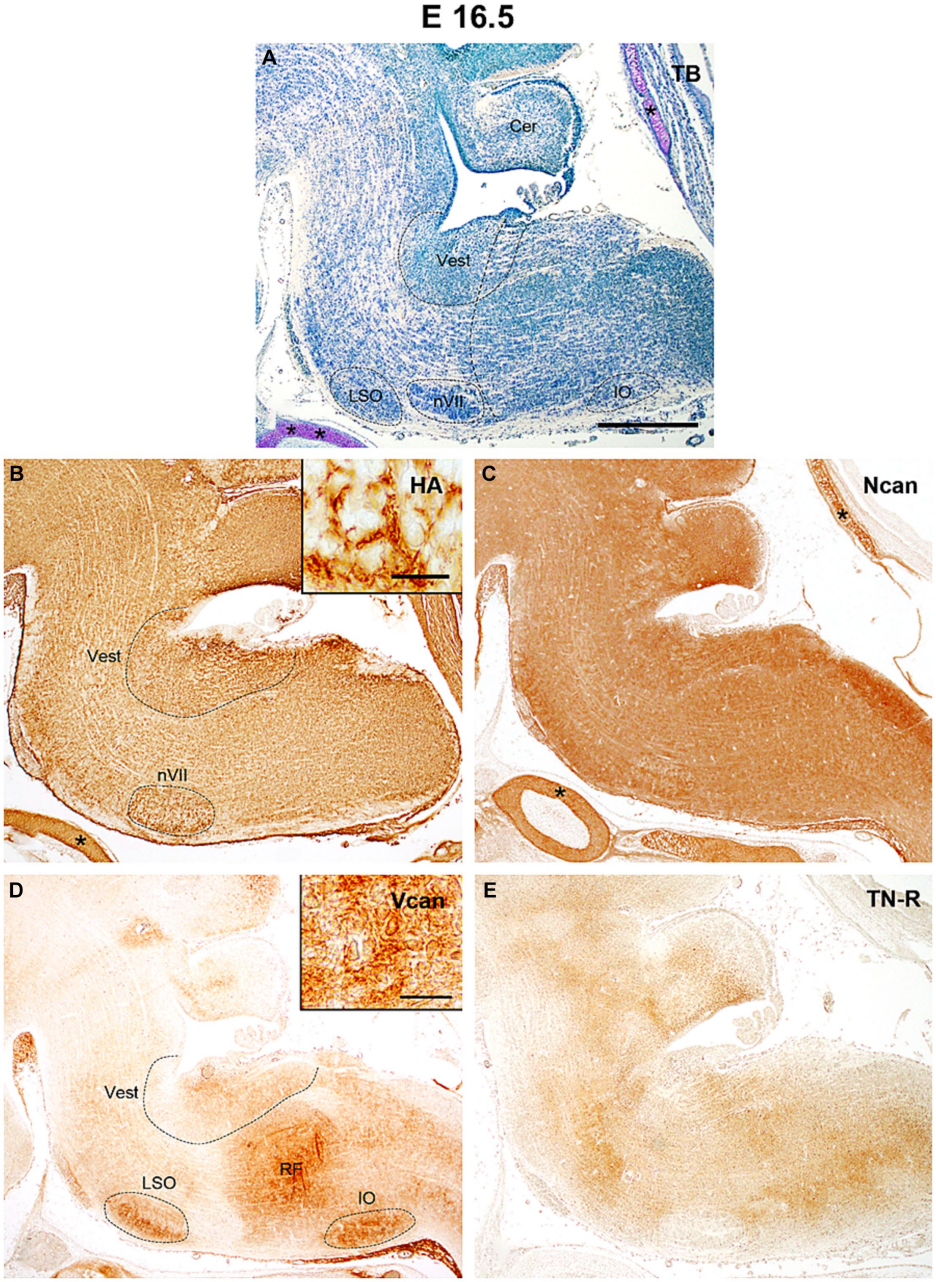
In the hindbrain of the E16.5 mouse embryo intense hyaluronic acid (HA) labeling was detected in the facial (nVII) and vestibular (Vest) nuclei **(B)**, while versican was accumulated in the reticular formation (RF), in the inferior olive (IO), lateral superior olive (LSO) and in the vestibular nuclei **(D)**. The neurocan (Ncan) and tenascin-R (TN-R) exhibited relatively homogenous labeling in the neuropil of the hindbrain **(C,E)**. Inserts in **(B)** and **(D)** show enlarged areas from the vestibular nuclei and reticular formation, respectively. The vertical dashed line in **(A)** shows the border between the pons and medulla oblongata. Asterisks indicate metachromasia **(A)** and labeled ECM molecules **(B,C)** in the cartilage of the skull. Cer, cerebellum; TB, toluidine blue. Scale bar 200 μm at low magnification and 20 μm at high magnification.

By E16.5 the rostral part of the hindbrain below the fourth ventricle enlarges to establish the pons whereas caudally the hindbrain gives rise to the medulla oblongata (Figure 5A). The level of hyaluronan remains about the same as before among the nuclei and besides the facial motor nucleus, it was also concentrated dorsally in the vestibular nuclei (Figure 5B). The neurocan still shows strong homogenous labeling which appears especially robust in the interstitium of the alar plate of the medulla (Figure 5C). The versican appeared in a relatively larger amount in the vestibular and olivary nuclei and the reticular formation but among these nuclei its staining intensity still stays at a relatively low level (Figures 4C,D, 5D). The staining intensities for tenascin-R change in different areas of the hindbrain resulting in a patchwork-like pattern but the darker areas do not correspond to the territories of any brainstem nuclei (Figure 5E).

**Figure 6:**
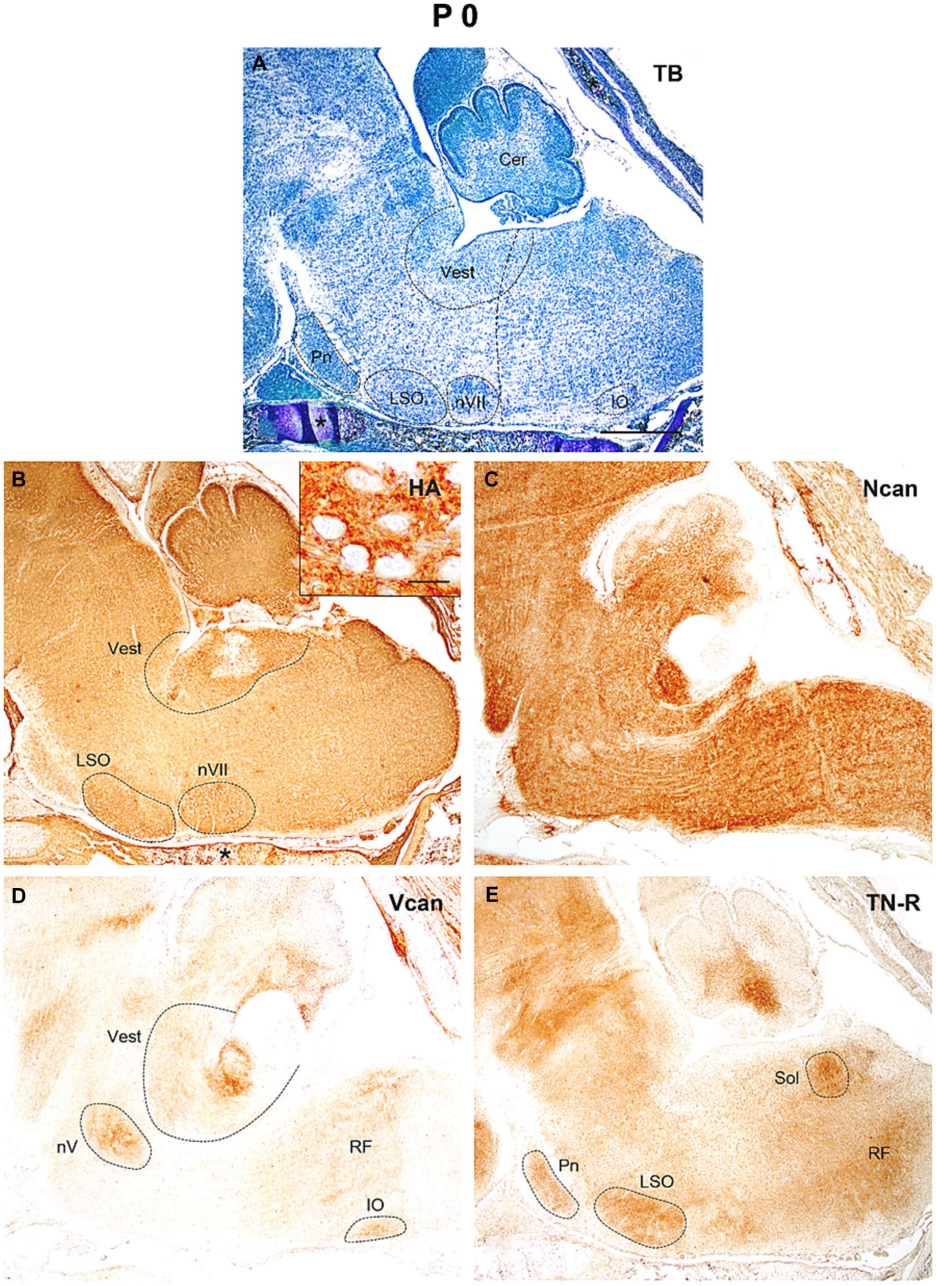
In the hindbrain of the newborn mouse (P0) the neuropil was strongly stained by hyaluronic acid (HA), neurocan (Ncan), and tenascin-R [TN-R, **(B,C,E)**], while versican (Vcan) labeled the neuropil in some brain stem nuclei **(D)**. Insert in **(B)** shows the enlarged area from the vestibular nuclei. The vertical dashed line in **(A)** shows the border between the pons and medulla oblongata. Asterisks indicate metachromasia **(A)** and labeled hyaluronic acid **(B)** in the cartilage of the skull. Cer, cerebellum; IO, inferior olive; LSO, lateral superior olive; nV, trigeminal motor nucleus; nVII, facial motor nucleus; Pn, pontine nuclei; RF, reticular formation; Sol, nucleus of the solitary tract; Vest, vestibular nuclei; TB, toluidine blue. Scale bar 200 μm at low magnification and 20 μm at high magnification.

At the time of birth (P0, Figure 6A) the neurocan was still evenly distributed in high concentration all over the hindbrain (Figure 6C), while the hyaluronan, versican, and tenascin-R give rise to higher staining intensities in the areas constituting the lateral superior and inferior olive, the trigeminal and facial motor nuclei, the vestibular as well as the pontine and solitary nuclei (Figures 6B,D,E).

We can summarize our results regarding the distribution of different ECM molecules during embryonic development in the hindbrain as follows. The hyaluronan was found in a high amount at E13.5 and stayed at this high level until the time of birth (Figures 4A,B). The neurocan also appeared in large quantity at E13.5 but it varied significantly between the different embryonic stages (Figure 4E). The versican showed relatively faint staining strength in the interstitium during the whole embryonic period but it reached high level within the nuclei of the brainstem (Figures 4C,D). The interstitial staining for tenascin-R showed a continuous increase and reached its maximum at the time of birth (Figure 4F).

### 3.2 Distribution of ECM molecules in the postnatal and adult hindbrain

All ECM molecules that were described in the embryo could be also detected in the early postnatal period at P4. However, contrary to the embryonic period and P0 the ECM molecules also appeared in the form of a perineuronal net.

#### 3.2.1 Distribution of ECM molecules in the nuclei and internuclear territories of the postnatal and adult hindbrain

**Figure 7:**
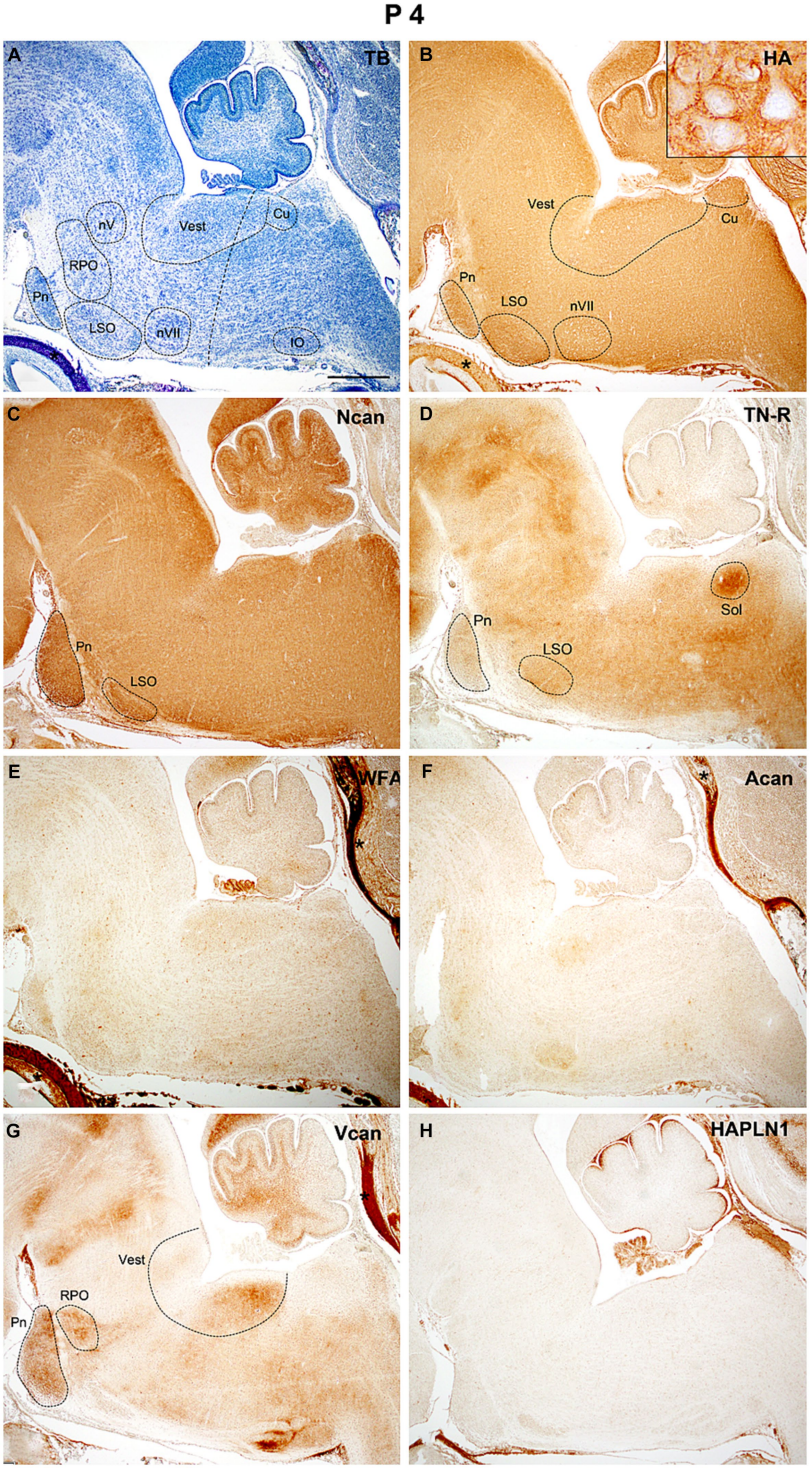
At the early stage of postnatal development (P4) thin positive perineuronal net appeared around the neurons in some brainstem nuclei [insert in **(B)**]. Strong internuclear and nuclear labeling was revealed with neurocan **(C)** and tenascin-R **(D)** antibodies. After birth, a weak diffuse reaction emerged with biotinylated *Wisteria floribunda* agglutinin **(E)** and aggrecan **(F)** antibodies which were not detected during embryonic development. The versican still produces labeling in certain nuclei of the hindbrain **(G)**, while no signals were found in slices treated with antibodies against hyaluronic acid and proteoglycan link protein 1 [HAPLN1, **(H)**]. Insert in **(B)** shows the concentration of hyaluronic acid around the vestibular neurons. Asterisks indicate metachromasia **(A)** and labeled ECM molecules **(B,E,F)** in the cartilage of the skull. The vertical dashed line in **(A)** shows the border between the pons and medulla oblongata. Cu, cuneate nucleus; IO, inferior olive; LSO, lateral superior olive; nV, trigeminal motor nucleus; nVII, facial motor nucleus; Pn, pontine nuclei; RPO, rostral preolivary region; Sol, the nucleus of the solitary tract; Vest, vestibular nuclei; TB, toluidine blue. Scale bar 200 μm at low magnification and 20 μm at high magnification.

At P4 (Figure 7A) both hyaluronic acid and neurocan showed intense staining in almost all areas of the hindbrain (Figures 7B,C). The neurocan signal was the strongest along the ventral side of the hindbrain including the pontine and lateral superior olivary nuclei (Figure 7C). Compared to the time of birth, the tenascin-R staining decreased all over the hindbrain (Figures 4F, 7D, 10E). The aggrecan could be first recognized at this time in our study and WFA staining was also detected earliest at P4, they appeared as weak homogeneous labeling in the interstitium of the hindbrain (Figures 7E,F). The versican labeling was still present whereas no reaction was found with the HAPLN1 antibody (Figures 7G,H).

**Figure 8:**
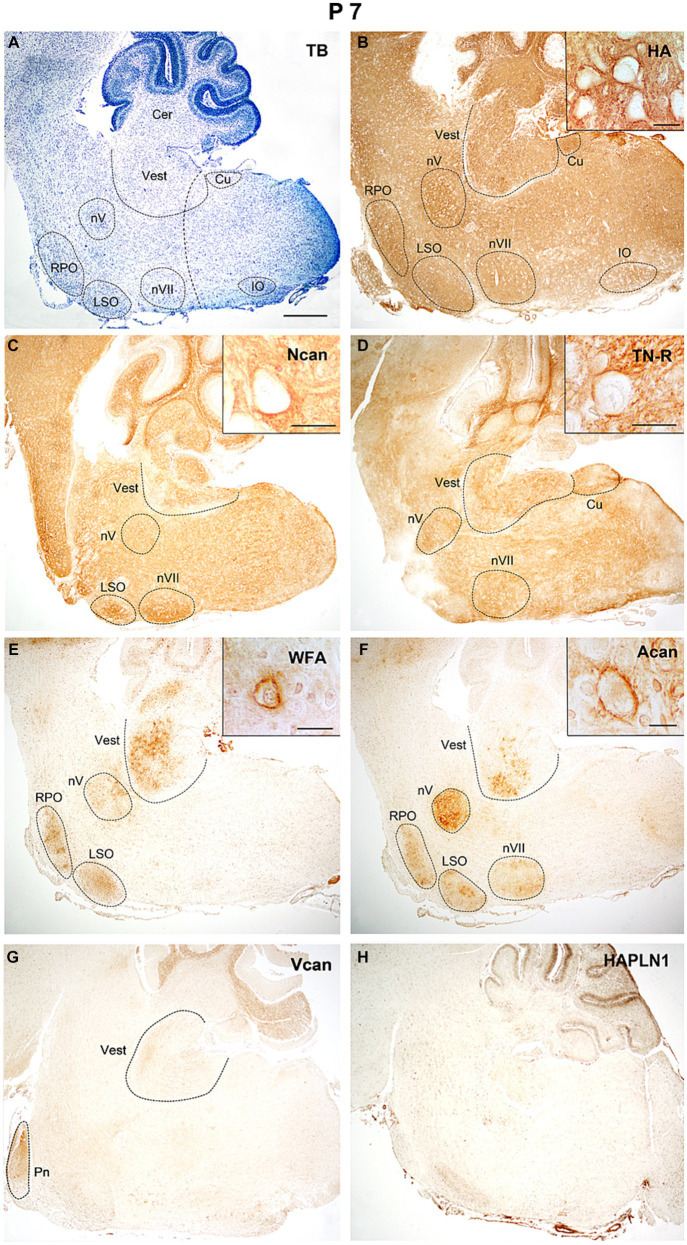
In the hindbrain of P7 mice hyaluronic acid, neurocan, and tenascin-R showed intense staining in all areas of the hindbrain **(B–D)**, while WFA and aggrecan signals were mainly concentrated in the nuclei **(E,F)**. The versican gave weak labeling in the pontine and vestibular nuclei, and the HAPLN1 is still not present in the hindbrain **(G,H)**. Inserts in **(B–F)** illustrate different ECM molecules in the perineuronal net around the cell bodies of neurons in the vestibular nuclei. The vertical dashed line in **(A)** shows the border between the pons and medulla oblongata. Cer, cerebellum; Cu, cuneate nucleus; IO, inferior olive; LSO, lateral superior olive; nV, trigeminal motor nucleus; nVII, facial motor nucleus; RPO, rostral preolivary region; Vest, vestibular nuclei; TB, toluidine blue. Scale bar 200 μm at low magnification and 20 μm at high magnification.

One week after birth (Figure 8A) the reaction for hyaluronan was still strong both in the nuclei and the internuclear territories (Figure 8B). The neurocan and tenascin-R showed strong staining intensities in the interstitium and also appeared in several nuclei of the hindbrain (Figures 8C,D). Except for some nuclei, the strength of the reaction for WFA and aggrecan was low all over the hindbrain (Figures 8E,F). The staining for versican decreased considerably (Figure 8G) and the HAPLN1 still could not be detected during this postnatal period (Figure 8H).

**Figure 9:**
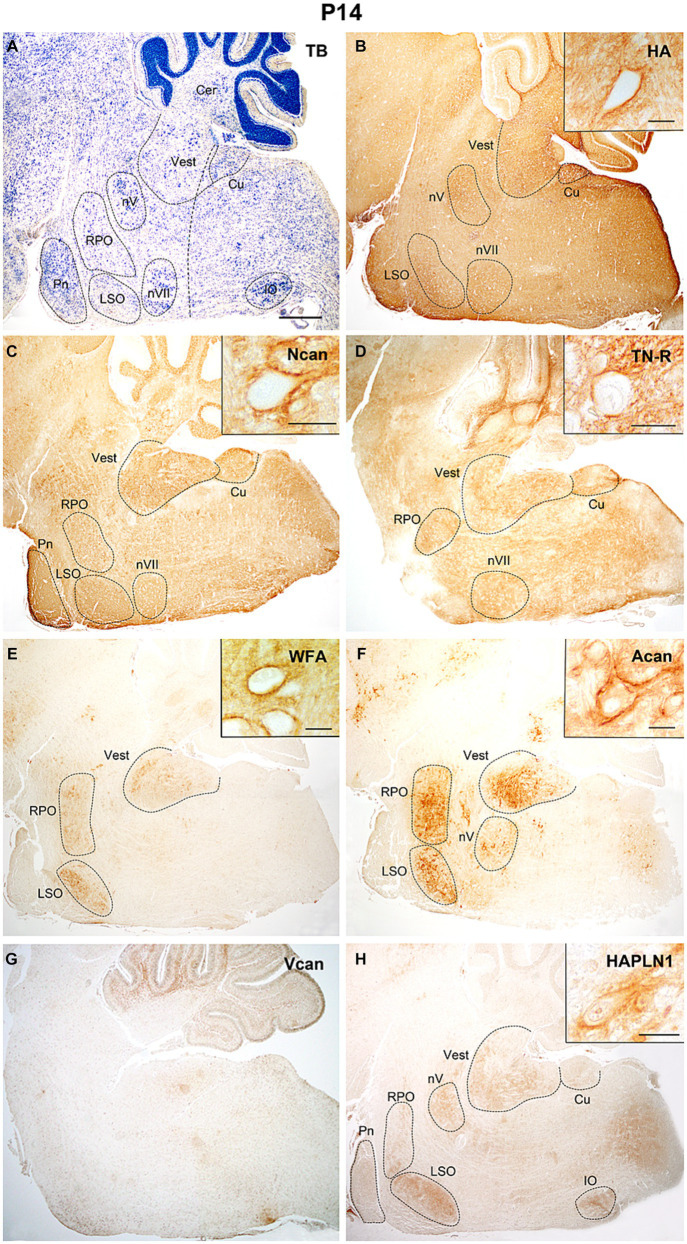
Two weeks after birth hyaluronic acid, neurocan, and tenascin-R showed strong neuropil labeling all over the hindbrain **(B–D)**. The WFA, aggrecan and hyaluronic acid, and proteoglycan link protein (HAPLN1) staining were localized in different brainstem nuclei **(E,F,H)**, while V0 and V1 forms of the versican disappeared in the hindbrain **(G)**. Inserts show cells surrounded by PNN labeled with different ECM molecules in the vestibular nuclei **(B–D)** and the lateral superior olive **(E,F,H)**. The vertical dashed line in **(A)** shows the border between the pons and medulla oblongata. Cer, cerebellum; Cu, cuneate nucleus; IO, inferior olive; LSO, lateral superior olive; nV, trigeminal motor nucleus; nVII, facial motor nucleus; RPO, rostral preolivary region, Vest, vestibular nuclei, TB, toluidine blue. Scale bar 200 μm at low magnification and 20 μm at high magnification.

**Figure 10:**
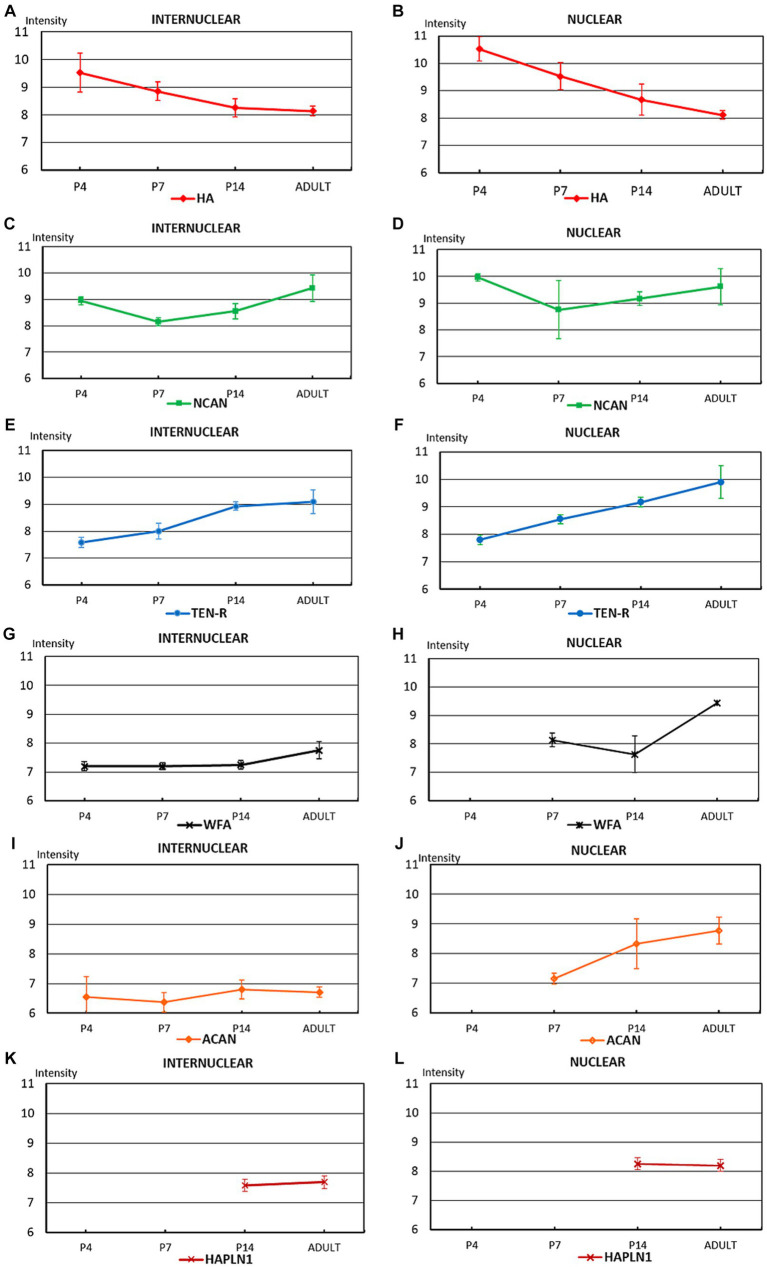
The diagrams summarize the optical intensities of ECM molecules in the internuclear areas **(A,C,E,G,I,K)** and different hindbrain nuclei **(B,D,F,H,J,L)** in the postnatal and adult hindbrains. The points indicate the average values, the bars correspond to the standard error of mean.

On the 14th postnatal day (Figure 9A), the investigated extracellular matrix molecules appeared in the vestibular and pontine nuclei, in the lateral superior and inferior olivary and cuneate nuclei, as well as in the rostral preolivary region and motor nuclei of the trigeminal and facial nerves (Figure 9). The hyaluronan decreased both in the nuclei and in other areas of the hindbrain (Figures 9B, 10A,B). The neurocan and tenascin-R reached the highest quantity during the studied postnatal periods (Figures 9C,D, 10C–F). The aggrecan and WFA labeling was mostly restricted in the labeled nuclei and its staining was still low in the interstitium (Figures 9E,F, 10G–J). The versican was no longer present and HAPLN1 was first detected in ECM of the postnatal hindbrain where it became visible both in the nuclei and the internuclear areas (Figures 9G,H).

**Figure 11:**
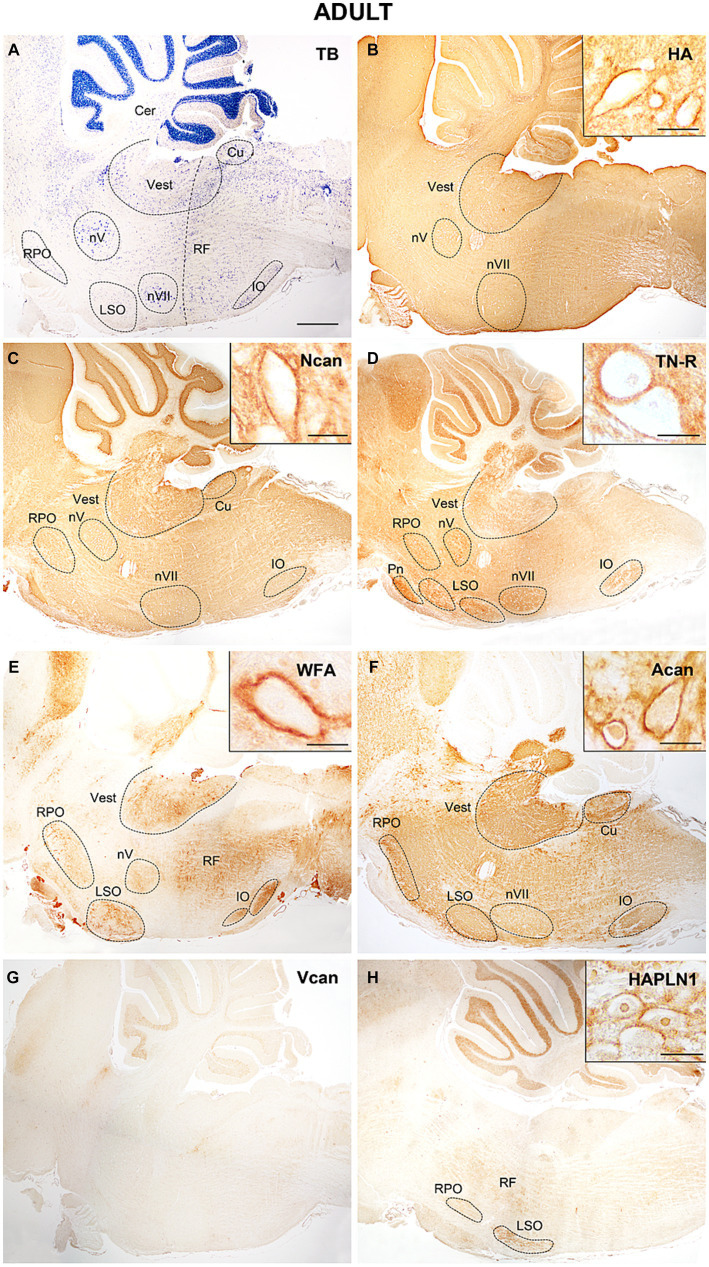
In adult mice hyaluronic acid presented a strong signal **(B)**, neurocan tenascin-R reached the highest concentration in the hindbrain **(C,D)**, while WFA and aggrecan showed lower staining intensities among the labeled nuclei **(E,F)**. The V0 and V1 forms of the versican were not detected in the adult hindbrain **(G)**, while HAPLN1 appeared in the interstitium and the perineuronal net **(H)**. Higher magnification images show intense staining in the perineuronal net of the vestibular nuclei. The vertical dashed line in **(A)** shows the border between the pons and medulla oblongata. Cer, cerebellum; Cu, cuneate nucleus; IO, inferior olive; LSO, lateral superior olive; nV, trigeminal motor nucleus; nVII, facial motor nucleus; RF, reticular formation; RPO, rostral preolivary region; Vest, vestibular nuclei; TB, toluidine blue. Scale bar 200 μm at low magnification and 20 μm at high magnification.

In the adult hindbrain (Figure 11A) the tenascin-R and neurocan produced high intensity values both within and outside of the labeled nuclei (Figures 10C–F, 11C,D). The staining with WFA and aggrecan achieved a relatively high level in the nuclei but they were kept at low values among the nuclei (Figures 10G–J, 11E,F). The staining level of hyaluronic acid seemed to be relatively low (Figures 10A,B, 11B). The HAPLN1 staining was kept at a constant level all over the hindbrain (Figures 10K,L, 11H) whereas the V1 and V0 forms of the versican disappeared from the brainstem (Figure 11G).

The temporal variation of the extracellular molecules during the postnatal period is illustrated in Figure 10. Between P0 and P4 the staining intensities of hyaluronan were kept about the same levels in the interstitium but its labeling was increased in the nuclei (Figures 4A,B, 10A,B). The staining intensities for neurocan decreased from P0 to P4 in the internuclear areas and it was first revealed in the nuclei at P4 (Figures 4E, 11C,D). The tenascin-R appeared at lower levels at P4 than P0 in the entire hindbrain (Figures 4F, 10E,F). The reaction with WFA and aggrecan was detected first at P4 at relatively low staining intensities (Figures 10G,J). Between the different postnatal periods, the level of hyaluronan decreased continuously both in the nuclei and in the internuclear areas of the hindbrain (Figures 10A,B). Contrary to the hyaluronan, the tenascin-R showed continuous growth (Figures 10E,F). The neurocan showed a deep decline between P4 and P7 but after that age, its staining pattern was changed similarly to the tenascin-R (Figures 10C,D). The WFA and aggrecan staining were kept at constant low values in the internuclear areas during the postnatal period of development (Figures 10G,I). In the nuclei, however, an abrupt increase could be detected between the P7 and the adult period for the aggrecan and after P14 also for the WFA staining (Figures 10H,J). The HAPLN1 was detected in the last investigated postnatal period (P14) when it appeared both in the nuclei and in the internuclear areas (Figures 10K,L). The staining intensity of HAPLN1 was kept at a steady level between P14 and adult animals.

#### 3.2.2 Distribution of ECM molecules in the perineuronal net of the postnatal and adult hindbrain

The neurons labeled by perineuronal net were chosen from six different nuclei including the somatomotor nuclei of the trigeminal and facial nerves, the vestibular nuclear complex, as well as the lateral superior olive and rostral preolivary region (Table 3).

**Table 3:** First appearance of extracellular matrix molecules in the perineuronal net of brainstem nuclei.

Nucleus	HA	Acan	Ncan	WFA	TN-R	HAPLN1
Trigeminal	P7	P7	P7	P7	P7	P14
Facial	P4	P7	P7	>P14	P7	>P14
Vestibular	P4	P7	P7	P7	P7	P14
Lateral superior olive	P4	P7	P7	P7	P7	P14
Rostral preolivary region	P7	P7	P14	P14	P7	P14

At P4 the perineuronal net was detected exclusively on the sections that were labeled with hyaluronic acid cytochemistry in the facial, vestibular, and lateral superior olivary nuclei, but the optical density of hyaluronic acid in the PNN seemed to be relatively low (Figure 7B). One week after birth the aggrecan, neurocan, tenascin-R, and WFA labeling also appeared in the perineuronal net in most of the investigated hindbrain nuclei (Figures 8C–F, Table 3). On the second postnatal week, the intensity value for hyaluronic acid, tenascin-R, and WFA increased while neurocan, and aggrecan decreased strongly. The HAPLN1 was detected for the first time in the perineuronal net with relatively low power in all examined hindbrain nuclei (Figure 9H, Table 3). In adult animals, the total intensity of PNN in the hindbrain nuclei was increased by about 40% compared to the last postnatal stage. The strongest labeling in the PNN comes for HAPLN1, tenascin-R, and neurocan. The HAPLN1 was mainly found in the motor and vestibular nuclei whereas the tenascin-R was located in the trigeminal nucleus, LSO, and RPO. Although the intensity values for HAPLN1 and Ncan immunoreaction increased considerably in the PNN of the adult animals, the tenascin-R and WFA produced similar values as we detected at P14.

In summary, except for the hyaluronan the ECM molecules begin to form a network around the somata of hindbrain neurons one week after birth. At this age, the PNN was dominated by aggrecan, neurocan, and tenascin-R. One week later, the aggrecan and neurocan intensities decreased more than 50%, and a moderate increase was found concerning the hyaluronan, tenascin-R, and WFA. The HAPLN1 was detected first at P14, and its values reached more the 250% growth in the adult hindbrain. Besides the HAPLN1, the WFA and hyaluronan also increased by about 50% during postnatal development while a strong increase was detected in the aggrecan.

## 4 Discussion

### 4.1 Spatial distribution of extracellular molecules in the embryonic and postnatal hindbrain

The development of the hindbrain begins after the closure of the neural tube when the rapid proliferation of the neuroepithelium results in prominent bulges in the proximal part of the rhombencephalon referred to as overt rhombomeres (r2-r6) giving rise to the pontine (r3-4) and the retro-pontine regions (r5-6) (Lumsden, 1990; Puelles et al., 2013). Caudal to the r6 the intersegment boundaries cannot be identified on the surface, but a hidden transverse organization was revealed resulting in five pseudo- or crypto rhombomeres (r7-11) giving origin to the medulla oblongata (Cambronero and Puelles, 2000; Tomas-Roca et al., 2016). Rostral to the overt rhombomeres the r1 and r0 or isthmus form the prepontine region where the segments can be separated with the help of molecular markers including Gbx2 and Fgf8 (Aroca and Puelles, 2005; Watson et al., 2019). Within each rhombomere, a particular overlapping expression pattern of the Hox family of homeodomain-containing transcription factors regulates the characteristic molecular environment for specific identity and developmental fates of the segment to produce distinct cell types and neuronal networks (Murphy et al., 1989; Prin et al., 2014). Besides their location along the longitudinal axis of the hindbrain, the fates of progenitors are also determined according to their dorsoventral positions provided by gradients of Sonigh hedgehog and Bone Morphogenic Protein secreted by the floor plate and roof plate, respectively (Briscoe et al., 1999; Jessell, 2000). It was supposed that the rhombomeres are transient structures necessary to the early development of the neural tube, but it seems that their Hox expression pattern is maintained until the perinatal and even in adult stages in the mouse (Farago et al., 2006).

The combination of Krox20 and Hoxa2 expression profiles with other genes induces the transformation of motoneuron progenitors into trigeminal motor neurons in the r2-3 rhombomeres at E9-10. After their birth in the ventricular zone, the cell bodies of these motoneurons move ventrally and then migrate laterally to establish the trigeminal motor nucleus at E12-13 (Jacob et al., 2001; Chandrasekhar, 2004). In our works the trigeminal nucleus could not be detected with ECM histochemistry in the embryo, it appeared first at birth by using a versican antibody (Figure 6D). At the end of the first week, the versican disappeared from the trigeminal nucleus but hyaluronan, neurocan, tenascin-R, and aggrecan became visible both in the interstitium and in the perineuronal net (Figures 7B–D,F), the HAPLN1 showed immunoreaction around the trigeminal motoneurons two weeks after birth (Figure 9H).

The facial branchiomotor neurons are born under the influence of Hoxa1, Hoxb1, and Hoxb2 homeotic genes in combination with other transcriptor factors in the r4 rhombomere between E9-11 and project their axons dorsally in the neural tube (Davenne et al., 1999; Jacob et al., 2001). The facial motoneurons begin a complex migration (Song, 2007): first, they live in the r4 and move caudally through the r5 rhombomere, after arriving at r6 they turn dorsally and then radially where they settle to form the facial motor nucleus at E12-14. In our experiments, the facial nucleus was revealed first with hyaluronan reaction at E15.5 (Figure 3B) and this ECM component was kept at a high level from this time until the adult stage. Besides hyaluronic acid, neurocan, tenascin-R, and aggrecan were also detected in the nucleus after the first postnatal week (Figures 8B–D,F).

The identity of vestibular neurons within the longitudinal columns of the alar plate is determined by successive actions of Hox genes from r2 rhombomeres to the caudal part of the hindbrain from E10 and the VNC could be first identified at E11.5 in the mouse hindbrain (Pasqualetti et al., 2007). After populating the rhombomeres, the majority of these neurons continue their development at the site of their generation. The subdivision of the vestibular nuclear complex (VNC) into different vestibular nuclei is based on their rhombomeric origin. In mice, the rostral rhombomeres give rise to neurons establishing the superior vestibular nucleus, the middle rhombomeric segments (r3-r5) produce the cells for the lateral vestibular nucleus, whereas progenitors from r5 to caudal cryptorhombomeres settle the medial and descending vestibular nuclei. According to the time of origin lateral to medial and superior to inferior gradients were observed in the settlement of the component of the VNC from E12 until E14 (Altman and Bayer, 1980; Maklad and Fritzsch, 2003; Lipovsek and Wingate, 2018). Our data showed that hyaluronan and versican were accumulated in the vestibular nuclei at the later stage of embryonic (Figures 5B,D) and in the early phases of postnatal development (Figures 6B,D). The perineuronal net was first recognized as the accumulation of hyaluronic acid around the vestibular neurons at P4 in our work (Figure 7B), similar time scale was detected in another study showing diffuse contour around vestibular neurons at P5 by using WFA labeling (Ma et al., 2019a,b). At P7 the ECM molecules produced intense staining in the interstitium and also in the perineuronal net of the VNC (Figures 8B–F), except for the HAPLN1 the distribution of different ECM molecules in the VNC resembles those of the adult form. Our data are in agreement with other studies showing that the critical period for the formation of perineuronal net falls between P3-7 when the connection between the afferents and vestibular neurons begins to function (Lai et al., 2006; Ma et al., 2019a,b).

The pontine nuclei constitute the majority of the mossy fiber input to the cerebellum. They begin to develop in the rhombic lip of the caudal hindbrain (r6-8) from E12.5. From the caudal rhombic lip, the pontine neurons undertake a long-distance migration in the anterior extramural stream: first, they move ventrally, then turn rostrally and migrate through r5-4 and go again ventrally to reach their final location on the sides of the floor plate in r3-4 (Kratochwil et al., 2017). After arriving at the ventral side of the neural tube between E14.5-E18.5, the pontine neurons settle their target regions according to their location which gives rise to distinct dorsoventral and anteroposterior subsets of neurons within the nucleus at the time of birth (Altman and Bayer, 1987a; Di Meglio et al., 2013). In our analysis, the different ECM molecules persisted below the detectable level during the whole embryonic development, therefore the pontine nuclei could not be delineated in the hindbrain with ECM immunohistochemistry. Postnatally, the tenascin-R was detected at birth (Figure 6E), and the other ECM molecules during the first week (Figures 7B–D,G). In the adult stage, only the tenascin-R could be seen (Figure 11D) whereas the level of the other ECM molecules decreased.

The inferior olivary nucleus gives origin to the climbing fiber input to the cerebellum. The cells for this nucleus originate from progenitors in the ventral area of the posterior rhombic lip in r6-8 at E9.0–10.5. The postmitotic cells migrate through the submarginal stream and establish a compact cell mass called olivary primordium close to the floor plate at E15. By E17 the cub-shaped cell group is divided by lamellae into three neuronal clusters called principal, dorsal, and accessory olive (Azizi and Woodward, 1987; Altman and Bayer, 1987b; Bourrat and Sotelo, 1990; Backer et al., 2007). Before birth, our results showed the position of the nucleus in the hindbrain with versican immunoreactivity at E16.5 (Figure 5D). After birth hyaluronan and HAPLN1 were detected in the nucleus at the end of the first and second week, respectively (Figures 8B, 9H), while almost all molecules appeared at relatively high levels in the adult animals (Figures 11C–F).

The lateral superior olive is the part of the superior olivary complex. As an important component of the ascending cochlear pathways, it is involved in the localization of sound. Via its efferent neurons, it is also responsible for the modulation of the excitability of the cochlear nerve and protection of the cochlea. The neurons of the LSO derive predominantly from the neuroepithelium in the r5 rhombic lip (Louvi et al., 2007; Maricich et al., 2009; Marrs et al., 2013), but its efferent neurons also come from the progenitor domain of visceral motor neurons in the basal plate of r4 (Bruce et al., 1997). Our works presented that the lateral superior olivary nucleus was intensively labeled with a juvenile form of the versican at E16.5 (Figure 5D) then hyaluronan and tenascin-R at the time of birth (Figures 6B,E). During postnatal development, the HA was kept at a high level in the nucleus until the adult stage (Figures 7B, 8B, 9B), whereas tenascin-R decreases after P4. The neurocan labeling appeared at P4 and remained within the nucleus in all postnatal stages (Figures 7C, 8C, 9C).

### 4.2 Temporal distribution of extracellular molecules in the embryonic and postnatal hindbrain

In the embryonic and early postnatal periods, the brain extracellular matrix is deposited in a relatively loose juvenile form constituting hyaluronan, neurocan, versican V0, and V1, as well as tenascin-C (Rauch et al., 1991; Milev et al., 1998). After P14 a mature denser network appears where the embryonic matrix molecules are exchanged by versican V2, aggrecan proteoglycans, tenascin-R, and HAPLNs. The expression of different proteoglycan molecules in the developing rat brains was investigated earlier with the help of radioimmunoassay, quantitative real-time PCR, and immunohistochemistry (Milev et al., 1998; Snyder et al., 2015).

In our samples, the hyaluronan appeared in a high amount at E13.5, showed constant intense staining in the interstitium of the embryo then reached a peak at P4 and decreased till the adult stage (Figures 4A, 10A). The HA was first detected in the nuclei at E15.5 and achieved the highest level at P4 when it was detected also in the PNN (Figures 4B, 10B). During postnatal development the hyaluronan level decreased by more than 30% in the nuclei (Figure 10B). Similarly to our data, hyaluronan was found in high concentration in the embryonic brain, reached a peak shortly after birth and was reduced to one-fourth of the postnatal level in the adult (Margolis et al., 1975). In the embryo, the HA forms a loosely packed network in the interstitium of the developing brain where it spreads into the synaptic cleft of the immature synapses to attenuate synaptogenesis (Wilson et al., 2020). During maturation of the synapses the hyaluronan, via its interaction with the CD44 receptor, initiates the formation of the postsynaptic density and stabilization of the synaptic contact (Wilson and Litwa, 2021). During postnatal development of the cerebellum, HA showed very weak diffuse labeling at P7 and displayed in the perineuronal net at P14 (Carulli et al., 2006).

The staining intensity for neurocan seemed to be kept at relatively constant high values during embryonic and postnatal development in the hindbrain, its level changed only about 15% in the interstitium between the different stages (Figures 4E, 10C). Despite its high embryonic level in the internuclear territories, the neurocan was revealed in the nuclei after birth (Figure 10D). In the rat brain, the neurocan was also detected in high concentration in the embryo, then reached a high peak by P10 and decreased abruptly during the second week of the postnatal period (Milev et al., 1998). Expression of neurocan in early periods of brain development and down-regulation during the postnatal period was also described in different cortical and subcortical areas of the brain (Köppe et al., 1997). After birth, the neurocan showed strong staining in the ECM in the cerebellar nuclei (Carulli et al., 2006) and the lateral geniculate nucleus (Sabbagh et al., 2018) during the first postnatal week then decreased after P14. The neurocan was first detected in the neuropil of pontine and vestibular nuclei at P14 and in the trigeminal nucleus at P21 by Brückner et al. (2000), whereas we found labeling for the neurocan in earlier developmental stages (P4) in the pontine and vestibular nuclei, and P7 in the trigeminal nucleus, respectively.

In our study, the V0 and V1 forms of the versican were found in the interstitium in all embryonic developmental stages and the first investigated postnatal week in the mouse hindbrain (Figures 4C,D, 7G, 8G). The amount of versican mRNA was the highest at E13.5 in the whole brain and accumulation of versican was detected in the periventricular region of the hindbrain by using immunohistochemistry (Snyder et al., 2015). The expression pattern of V0 and V1 forms of the versican in the rat brain was found to be similar to the neurocan but it appeared at a higher quantity in the embryo (Milev et al., 1998).

In our results aggrecan was first detected after birth and it was kept at a low level in the internuclear areas of the postnatal hindbrains (Figure 10I). In the nuclei the aggrecan labeling increased by more than 20% between P7 and the adult stage (Figure 10J). At P7 the aggrecan was also incorporated into the PNN but its level decreased more than half until the adult. Similarly to our data, the aggrecan could not be revealed in the embryonic brain with the help of immunohistochemical methods (Zimmermann and Zimmermann, 2008), and its mRNA was not expressed in the brain and spinal cord before E19 (Matthews et al., 2002). In another study however, the aggrecan was found in low concentration in the embryo, and its level was raised twice by the time of birth and during the first two weeks of postnatal development (Milev et al., 1998). The postnatal changes of aggrecan showed a similar distribution in the hippocampus and parietal cortex to our nuclear labeling between P7 and P14 (Zimmermann and Zimmermann, 2008). In the early postnatal stages, the aggrecan was not detected in the deep cerebellar nuclei (Carulli et al., 2007) or appeared as weak labeling in the cells of the medial nucleus of the trapezoid body (Schmidt et al., 2020) and in the lateral geniculate nucleus (Sabbagh et al., 2018).

The *Wisteria floribunda* agglutinin is a lectin that labels selectively N-acetylgalactosamine residues of glycoproteins and has been routinely used to reveal the perineuronal net around the neurons (Matthews et al., 2002). It is widely believed that WFA lectin mostly detects the side branches of the aggrecan, although the exact glycan motif is unknown (Miyata and Kitagawa, 2017; Fawcett et al., 2019). In our specimens, neither WFA nor aggrecan labeling could be registered in the embryonic hindbrain. Both of them appeared in the interstitium at P4 (Figures 7E,F) and were accumulated in the nuclei at P7 (Figures 8E,F). In the nuclei the strength of WFA staining was increased by about 20% in the adult hindbrains compared to P7 (Figure 10H) and WFA labeling showed a continuous increase in the PNN during postnatal development.The staining level of aggrecan was below WFA which may indicate that WFA should bind to the carbohydrate moieties of other chondroitin sulfate ECM components such as neurocan, or brevican (Härtig et al., 2022).

The tenascin-R was found in the hindbrain at E15.5 (Figure 3E). It showed a relatively weak diffuse labeling in the hindbrain during the whole embryonic development where its immunoreaction was increased by about 20% by the time of birth (Figure 4F). During the postnatal stages the level of tenascin-R was continuously increased in the hindbrain outside in inside the nuclei until the adult stage (Figures 10E,F). Similar temporal distribution for tenascin was reported in the mouse cortex where it was first detected around E16, and its level was increased until the third postnatal week (Wen et al., 2018). In the lateral geniculate nucleus, the level of tenascin-R was increased from P3 to P12 and then decreased until P25 (Sabbagh et al., 2018).

The HAPLN1 was revealed in the hindbrain in the last stage of the developmental periods we examined (Figure 9H). Although its intensity level was not changed too much in the interstitium and nuclei of the hindbrain at P14 and in the adult animals, its amount was increased more the three-folds in the PNN of the neurons between the same stages (Figures 10K,L). In the medial and lateral nuclei of the trapezoid body, HAPLN1 presented a faint immunoreactivity at P7 but its labeling intensity increased systematically between P14 and P28 (Sabbagh et al., 2018; Schmidt et al., 2020). With the help of proteomic analyses, HAPLN1 was revealed at P3 in very low concentration in the visual thalamus but its amount was abruptly increased at P12 (Sabbagh et al., 2018).

### 4.3 Formation of perineuronal net in the postnatal hindbrain

In the interstitium of the embryonic brain and spinal cord, the extracellular matrix molecules establish a loose meshwork whose chief components are the hyaluronic acid, neurocan, juvenile (V0 and V1) forms of versican as well as tenascin-C (Guldfinger et al., 2010; Frischknecht and Happel, 2016). In the developing brain, these molecules control the proliferation and differentiation of neurons and glial cells, as well as migration, axonal pathfinding, and synaptogenesis of neurons. During postnatal development, new chondroitin sulfate proteoglycans such as aggrecan and brevican are upregulated in the ECM, while tenascin changes its molecular structure into the form of tenascin-R. As a consequence of these molecular changes, the relatively loose, embryonic ECM becomes a netlike insoluble complex in the late postnatal and adult brains which inhibits regeneration and reorganization processes. Not only the molecular constitution of the ECM but also the sulfation pattern of the glycosaminoglycans within the chondroitin sulfate proteoglycans undergo deep changes after birth: in the embryonic brain the GAGs are mostly 6-sulfated, whereas in the adult CNS only 2.5% of the GAGs are 6-sulfated and more the 90% are sulfated in the 4 positions (Nadanaka et al., 1998 Fawcett et al., 2019). The sulfation pattern of GAGs plays a pivotal role in the functional properties of CSPGs, it determines, for example, the promoting or suppressing properties of the ECM to axon growth or plasticity.

During postnatal development of the brain, the overwhelming majority of the ECM macromolecules remain in the parenchyma as an interstitial matrix but about 2% of them establish a very condensed and stable network around cell bodies, proximal dendrites and axon initial segments of the neurons as perineuronal net or enwrap the node of Ranvier as perinodal matrix (Fawcett et al., 2019). We found the first sign of accumulation of the extracellular matrix in the form of aggregates of hyaluronan around the neurons in the hindbrain on the fourth postnatal day (Figure 7B). Our data that HA could be detected as the first component of PNN are in agreement with the generally accepted view that the hyaluronic acid establishes the main structural component of the perineuronal net (Bosiacki et al., 2019; Carulli and Verhaagen, 2021), and most CSPGs are bound to the cell surface via hyaluronic acid. Accumulation of hyaluronic acid was accompanied by incorporation of different proteoglycan macromolecules and tenascin-R into the PNN during the first and second postnatal weeks in different nuclei of the hindbrain (Figures 8, 9). The formation of PNN was completed by upregulation of HAPLN1 at P14 (Figure 9H) which stabilizes the PNN by linking the proteoglycan aggregates to the hyaluronan backbone (Fawcett et al., 2019).

Although the formation of the perineuronal net was investigated in the different areas of the brain, only a limited number of studies were concerned with describing the formation of PNNs around different neurons of the brainstem (Köppe et al., 1997; Brückner et al., 2000; Schmidt et al., 2020). By using WFA staining and CSPG-immunoreactivity Köppe et al. (1997) detected weak immature PNN in different brainstem regions between the first and second postnatal weeks while the strong adult-like staining pattern of PNN was attained in the third one. In this work, WFA labeling appeared around the cell bodies of the trigeminal motor nucleus and in the vestibular nucleus at the second postnatal week, whereas we found weak staining for WFA and different CSPGs in the same nuclei one week earlier (Figures 8C,E,F). But our results are in agreement with Brückner et al. (2000), who also found that antiserum to chondroitin sulfate proteoglycans showed faintly stained PNN in the trigeminal and vestibular nuclei in the first postnatal week and produced fully developed labeling at P14. WFA-positive PNN was first described at P5 around GABAergic neurons in the superior vestibular nucleus, while consolidated PNNs were observed from P7 to P9 in the other vestibular nuclei. The number of PNN-bearing cells reached the adult number by P21 when the cells were mostly surrounded by an adult-like form of the net (Ma et al., 2019a,b). According to these works and our study, the PNNs in the different nuclei of the brainstem could be detected between the first and second postnatal weeks usually as faintly stained immature structures which was fully developed after P14.

Besides the brainstem, the constitution of PNN was also studied in the developing deep cerebellar nuclei, and Golgi, Purkinje, and granule cells of the cerebellar cortex by using histochemistry and *in situ* hybridization (Carulli et al., 2007). Similarly to the results in the hindbrain, WFA labeling was first detected in the PNN at P7, and the HAPLN1 displayed from P14, but aggrecan and neurocan appeared one week later in the deep cerebellar nuclei. In the cerebral cortex and hippocampus, the perineuronal net was mostly associated with GABAergic parvalbumin-containing inhibitory neurons and to a lesser extent with pyramidal neurons (Carulli and Verhaagen, 2021). In different areas of the forebrain net-like structures were detected with WFA and CSPGs at P14 and they reached their adult-like structures from P21 to P40 but the exact time of termination of PNN formation could be strongly modulated under environmental factors (Köppe et al., 1997; Brückner et al., 2000; Miyata and Kitagawa, 2017).

In different brain areas, the conversion between the juvenile and adult forms of ECM and the formation of the perineuronal net coincided with the end of the critical period. The critical period is defined as an interval where experience from the environment is necessary to shape and refine the neuronal circuits involved in processing the sensory information (Cisneros-Franco et al., 2022). The critical periods were thoroughly investigated in the visual and auditory cortex (Pizzorusso et al., 2002; de Villers-Sidani et al., 2010; Lee et al., 2017), and both studies showed that the closure of the critical periods coincided with the formation of PNNs around GABAergic neurons in V1 and A1, respectively. After the critical period, the incoming sensory stimulus is not able to reshape the cortical circuits which gives the morphological background to process the information within the cortex in a reliable manner (Cisneros-Franco et al., 2022). Activity-dependent formation of the perineuronal net was also described in the barrel cortex of rats and mice where the first month of life seemed to be critical for the formation of PNN around parvalbumin-expressing cells (Mcrea et al., 2007) and in the basolateral amygdala where the formation of PNN protects fear memories from erasure (Gogolla et al., 2009; Hylin et al., 2013; Xue et al., 2014).

It was described that the maturation of negative geotaxis in the vestibular area of the brainstem coincided with the consolidation of PNN around GABA-ergic interneurons in the vestibular nuclei at P9 when the connections between vestibular afferents and neurons became functional (Ma et al., 2019a,b). The connections between the vestibular afferents with otolith-related vestibular neurons become functional by the end of the first postnatal week when vestibular neurons can detect vertical linear acceleration (Lai et al., 2006). In agreement with these results, we detected strong PNN labeling composed of HA and CSPGs around the vestibular neurons about the same time scale (Figures 8B,C,E,F).

## 5 Conclusion

For the time being this study is the only comprehensive work on the ECM expression in the developing brainstem. Using histochemistry and immunohistochemistry, we have described the spatial and temporal distribution of the major ECM molecules in the neuropil and perineuronal net in the embryonic, postnatal and adult hindbrain of the mice. Our results showed differences in the spatial and temporal distribution of the ECM expression pattern in the developing hindbrain. Until the time of birth hyaluronic acid and versican were present in the internuclear and nuclear areas, whereas neurocan and tenascin-R were only detected in the internuclear areas. Four days after birth, all ECM molecules examined here were found in both areas, nevertheless only HA, TN-R and neurocan showed further continuous expression during the period studied. The early disappearance of versican indicates the changing of the juvenile matrix into the adult form. Expression of HAPLN1 along with the elevation of aggrecan, TN-R and neurocan in the nuclei parallel with the formation of the perineuronal net may indicate the stabilization of the synaptic contacts and the reduction of neural plasticity.

## Data availability statement

The original contributions presented in the study are included in the article/supplementary material, further inquiries can be directed to the corresponding author.

## Ethics statement

The animal study was approved by the study protocol was reviewed and approved by the Animal Care Committee of the University of Debrecen, Hungary according to national laws and EU regulations [European Communities Council Directive of 24 November 1986 (86/609/EEC)] and was properly carried out under the control of the University’s Guidelines for Animal Experimentation (license number: 11/2011/DEMAB). The study was conducted in accordance with the local legislation and institutional requirements.

## Author contributions

IW: Data curation, Investigation, Methodology, Visualization, Writing – original draft, Writing – review & editing. AD: Data curation, Formal analysis, Software, Writing – original draft, Writing – review & editing. ZM: Funding acquisition, Investigation, Methodology, Supervision, Writing – original draft, Writing – review & editing. CM: Conceptualization, Investigation, Supervision, Validation, Writing – original draft, Writing – review & editing. AB: Conceptualization, Data curation, Investigation, Supervision, Visualization, Writing – original draft, Writing – review & editing.

## Footnotes

^1^http://developingmouse.brain-map.org

^2^imagej.nih.gov

## Glossary

Acan: aggrecan
bHABP: biotinylated hyaluronan binding protein
BSA: bovine serum albumin
Cer: cerebellum
CSPG: chondroitin sulfate proteoglycans
Cu: cuneate nucleus
DAB: 3,3 diaminobenzidine-tetrahydrochloride
E: embryonic
ECM: extracellular matrix
HA: hyaluronic acid
HAPLN1: hyaluronan and proteoglycan link protein 1
IO: inferior olive
LSO: lateral superior olive
MH: medullary hindbrain
nV: trigeminal motor nucleus
nVII: facial motor nucleus
Ncan: neurocan
NHS: normal horse serum
NGS: normal goat serum
NRS: normal rabbit serum
P: postnatal
PB: phosphate buffer
PH: pontine hindbrain
PMH: pontomesencephalic hindbrain
Pn: pontine nuclei
PNN: perineuronal net
PPH: prepontine hindbrain
RPO: rostral preolivary region
RF: reticular formation
Sol: nucleus of tractus solitarius
TB: toluidine blue
TN-R: tenascin-R
Vcan: versican
Vest: vestibular nuclei
WFA: *Wisteria floribunda* agglutinin
